# Status of Agents Targeting the HGF/c-Met Axis in Lung Cancer

**DOI:** 10.3390/cancers10090280

**Published:** 2018-08-21

**Authors:** Oshin Miranda, Mariya Farooqui, Jill M. Siegfried

**Affiliations:** 1 Department of Pharmacology and Masonic Cancer Center, University of Minnesota, Minneapolis, MN 55455, USA; miran129@umn.edu (O.M.); faroo001@umn.edu (M.F.); 2 Department of Pharmacology, University of Minnesota, 321 Church Street SE, 6-120 Jackson Hall, Minneapolis, MN 55455, USA

**Keywords:** hepatocyte growth factor (HGF), c-Met (mesenchymal epithelial transition factor or hepatocyte growth factor receptor), targeted therapy, lung cancer

## Abstract

Hepatocyte growth factor (HGF) is the ligand for the tyrosine kinase receptor c-Met (Mesenchymal Epithelial Transition Factor also known as Hepatocyte Growth Factor Receptor, HGFR), a receptor with expression throughout epithelial and endothelial cell types. Activation of c-Met enhances cell proliferation, invasion, survival, angiogenesis, and motility. The c-Met pathway also stimulates tissue repair in normal cells. A body of past research shows that increased levels of HGF and/or overexpression of c-Met are associated with poor prognosis in several solid tumors, including lung cancer, as well as cancers of the head and neck, gastro-intestinal tract, breast, ovary and cervix. The HGF/c-Met signaling network is complex; both ligand-dependent and ligand-independent signaling occur. This article will provide an update on signaling through the HGF/c-Met axis, the mechanism of action of HGF/c-Met inhibitors, the lung cancer patient populations most likely to benefit, and possible mechanisms of resistance to these inhibitors. Although c-Met as a target in non-small cell lung cancer (NSCLC) showed promise based on preclinical data, clinical responses in NSCLC patients have been disappointing in the absence of *MET* mutation or *MET* gene amplification. New therapeutics that selectively target c-Met or HGF, or that target c-Met and a wider spectrum of interacting tyrosine kinases, will be discussed.

## 1. Biology of c-Met and Its Ligand, HGF 24pt

The c-Met oncogene was first isolated from a human osteosarcoma cell line, which contained a DNA rearrangement: the translocated promoter region (TPR) locus on chromosome 1 was fused to the *MET* gene on chromosome 7 [1]. The *MET* gene produces a protein that is a tyrosine kinase receptor. The c-Met receptor, whose only known ligand is hepatocyte growth factor (HGF) [2], exists as a disulfide-linked heterodimer of the α and β chains, which forms upon proteolytic cleavage of the c-Met precursor [1]. The protein contains an extracellular domain for ligand binding, a membrane spanning domain, a juxtamembrane portion, the catalytic kinase domain, and a C-terminal docking site [3]. In the tumor microenvironment, growth factors and cytokines are frequently secreted that are capable of activating or further enhancing metastasis by developing motility and invasiveness to the tumor cells. Hepatocyte growth factor (HGF), the ligand for c-Met, was identified as a secreted factor responsible for enhancement of motility and invasion, that also caused cell scattering [2]. HGF in the tumor microenvironment can be derived from either the tumor cells or the tumor-associated stromal cells [2], and in lung cancer is mainly produced by the mesenchymal cells in the stroma. HGF is primarily a paracrine factor produced by mesenchymal cells and fibroblasts. Under special circumstances, such as hypoxia, cancer epithelial cells can secrete HGF [3].

HGF, such as the c-Met receptor, is produced in an inactive state and then converted into its active form via proteolysis. The active state of HGF consists of four Kringle domains (K1–K4), an amino (N) domain and a serine protease homology domain (SPH), whose interactions facilitate receptor dimerization [4]. The binding of active HGF to c-Met leads to oligomerization of receptor, activation of the catalytic portion, tyrosine residue autophosphorylation, and docking of substrates, causing activation of downstream signaling processes [5,6]. 

Binding of HGF to c-Met leads to autophosphorylation on the tyrosine residues Y1234 and Y1235 at the tyrosine kinase domain, activating further autophosphorylation of Y1349 and Y1356 residues near the COOH terminus. This activates the phosphotyrosine multifunctional docking site, which recruits intracellular adapters through Src and activates downstream signaling events [7]. Another important effect of HGF-mediated activation of c-Met is the stimulation of downstream effectors through the RAS/mitogen-activated protein kinase (MAPK) signaling pathway [8]. The HGF/c-Met pathway is also modulated by other proteins such as integrins which work as a platform that promotes the activation of RAS and PI3K, plexin B1, semaphorin and the death receptor Fas [9]. A number of biological activities such as cell proliferation, cell survival, motility function and morphogenesis are triggered by c-Met downstream signaling through these second messengers [6,7].

It is also well-established that activation of other tyrosine kinases participate in maximizing HGF/c-Met effects. The epidermal growth factor receptor (EGFR) plays a paramount role in potentiating c-Met–mediated cell proliferation, cell invasion and cell survival [10]. EGFR activation can cause a Src-dependent activation of c-Met that is ligand independent [11]. Likewise, downstream of c-Met activation, PGE_2_ release occurring after COX2 induction can increase activity of matrix metalloproteinases that release EGFR ligands such as amphiregulin [12]. EGFR and c-Met can have a synergistic effect to advance the malignant phenotype [13,14]. Other oncogenic mechanisms work to enhance c-Met action. For example, c-Met along with insulin-like growth factor 1 receptor can synergistically increase cell invasion and cell migration in cancer cells [15]. RAS protein in its activated form induces c-Met expression through a positive feedback mechanism [16]. Hypoxia is also known to positively regulate c-Met activity via tumor angiogenesis [17]. A complex system of reinforcing interactions modulate and govern the magnitude and duration of c-Met signaling in the cell.

## 2. HGF/c-MET Axis in Non-Small Cell Lung Cancer

Generally, activation of c-Met by HGF is controlled through release of ligand by a paracrine process in which mesenchymal cells and cells of the innate immune system secrete HGF, followed by ligand activation on the cell surface and internalization of the activated receptor. In embryonic development, the HGF/c-Met system is active as organs are forming, but is largely silent in the adult, unless triggered during wound healing. In a variety of cancers however, the HGF/c-Met pathway is constitutively activated. The mechanism of activation include gene amplification, over-expression of the c-Met and/or HGF proteins, increased cross-signaling between c-Met and other tyrosine kinases, and MET gene mutation. Amplification of the MET gene has been found in a number of solid tumor types, including gastric cancer, where sensitivity to a c-Met tyrosine kinase inhibitor was high, and the c-Met pathway was important in maintaining cell survival [18]. Cancer cells containing MET gene amplification were highly dependent on c-Met signaling for both proliferation and cell survival.

c-Met overexpression often occurs in the absence of gene amplification. In breast cancer, c-Met overexpression was an independent predictor of aggressive malignancy with poor patient survival [19]. Overexpression of c-Met protein is also commonly found in NSCLC tissues [20]; several comparative studies detected c-Met overexpression in 60% of cases, while phospho- c-Met was elevated in 40–100% cases [21,22]. Increased protein expression, as compared to the level measured in normal tissues, has been observed in multiple neoplasms, and the degree of protein overexpression is often related to stage and extent of tumor progression [21,22]. c-Met overexpression was associated with advanced stage of disease, poor outcome and poor survival rates in lung and breast cancer [23,24,25].

A rare mechanism that leads to c-Met activation is activating mutations. Both missense germ line mutations in the tyrosine kinase domain and rare sporadic mutations have been detected in less than 1% of renal carcinoma, melanoma, small-cell lung carcinoma and mesothelioma [26]. Oncogenic mutations are found outside the kinase domain, such as mutations in the semaphorin domain (E168D, L229F, S323G, and N375S) and the juxtamembrane domain (R988C, T1010I, S1058P, and exon 14 deletions) of NSCLC cells [26]. The phosphorylation of Y1003, located in the juxtamembrane domain, is responsible for internalization of the c-Met receptor by association with the CBL (Casitas B-lineage Lymphoma) ubiquitin ligase. When there is a deletion of exon 14, the loss of Y1003 leads to c-Met accumulation on the cell surface and high HGF stimulation contributes to cancer progression [26]. Although semaphorin domain and juxtamembrane domain c-Met mutations develop at a low frequency (about 4% of NSCLCs) they provide proof of the oncogenic capacity of this axis.

## 3. HGT/c-Met Axis in Small Cell Lung Cancer (SCLC)

Accumulating evidence shows that activation of the HGF/c-Met pathway in SCLC cells also leads to increased tumor growth and survival. Many small cell tumors have increased plasma levels of HGF and SCLC can also contain *MET* amplification. In an in vivo model, c-Met inhibitors such as crizotinib and golvatinib arrested the cell cycle and led to decreased SCLC cell growth and metastasis. This indicates that some SCLC may be sensitive to inhibition of the c-Met pathway. *MET* amplification was also shown to promote resistance towards anti-cancer drugs in SCLC [27]. In an orthotopic model, c-Met inhibitors arrested metastasis in SCLC cells with elevated HGF levels [28]. A recent report found activation of the c-Met pathway in chemoresistant or chemorelapsed SCLC cell lines, which occurred through increased HGF levels and increased *MET* gene amplification. Inhibition of c-Met caused anti-tumor effects on these chemoresistant SCLC cell lines both in vitro and in vivo. Thus HGF/c-MET-mediated signaling may be important in growth and progression of SCLC [29].

## 4. Therapeutics to Inhibit the HGF/c-Met Axis

The HGF/c-Met axis has been targeted in several ways for potential cancer treatment, including targeting receptor activation and ligand binding. Multiple agents (small molecule tyrosine kinase inhibitors [TKIs] of c-Met and antibodies directed against either the c-Met protein or HGF) have completed or are currently in clinical trials. Active clinical trials targeting this pathway, including new investigational agents, are summarized in Table 1. Despite the common finding that the HGF/c-Met axis is overactive in many NSCLC, results of most completed clinical trials in patients without genetic alterations in the MET gene were disappointing, with few objective responses, even in combination therapy trials. Patients with amplification or mutation of the MET gene showed high response rates to HGF or c-Met targeting, suggesting these genetic changes are associated with c-Met pathway addiction, which is needed for clinical response to agents tested to date. Results of completed trials and ongoing clinical testing of the agents in Table 1 will be discussed by the mechanism of action below.

## 5. Crizotinib: First Generation c-Met Tyrosine Kinase Inhibitor

Synthetic small-molecule c-Met TKIs are low molecular weight molecules that compete for the adenosine triphosphate (ATP) binding site of the c-Met tyrosine kinase domain [20]. This prevents activation of the receptor and arrests downstream signaling. Several small molecules that target c-Met show a lack of specificity and can also impede the ATP binding site in other kinases such as vascular endothelial growth factor receptor (VEGFR) and the translocated EML4-anaplastic lymphoma kinase (ALK). Crizotinib (PF-02341066) is a compound developed to target c-Met that also showed a high affinity for the translocated ALK fusion protein. In preclinical studies, crizotinib successfully suppressed NSCLC cell growth, migration and cell survival in models that expressed c-Met [13,20]. This compound has shown efficacy at well tolerated doses in NSCLC patients in patients with amplified or mutated c-Met, and in those with ALK abnormalities, but had little activity in NSCLC without these abnormalities [8]. In a phase III trial of first-line treatment in ALK (Anaplastic Lymphoma Kinase) Positive East Asian NSCLC with an ALK translocation or inversion, crizotinib was found to improve progression free survival (median 11.1 months), as compared to chemotherapy (median 6.8 months) [38] which led to its rapid FDA approval for ALK positive disease.

## 6. Tyrosine Kinase Inhibitors with Selectivity for c-Met

Volitinib (Savolitinib) is a selective c-Met inhibitor that blocks c-Met activity in an ATP-dependent manner. It has been shown to have anti-tumor activity in gastric and papillary renal carcinoma [39,40] in xenograft models. More recently in preclinical studies, it has been reported to inhibit tumor growth in NSCLC by blocking the PI3K/AKT, MAPK signaling and c-Myc down regulation [41]. Intravenous delivery of volitinib in gastric cancer models with amplification of the MET gene showed dose-dependent tumor regression [39]. Volitinib is being tested in combination with gefitinb in EGFR mutant NSCLC (Table 1). 

SAR125844: This derivative of triazolopyridazine was first identified as a selective inhibitor of both wild type c-Met and c-Met with kinase domain mutations in gastric cancer cell lines. Xenograft studies with MET amplified gastric tumor cells showed significant tumor growth inhibition because of antiproliferative and proapoptotic effects of the drug and down regulation of PI3K/AKT and RAS/MAPK pathways [42]. Pharmacokinetics studies were performed and analyzed in several species from mice to dog [43]. Phase I dose escalation and dose expansion study in patients with advanced tumors showed modest antitumor response in patients with MET amplified gastric cancers at 570 mg/m^2^ and was well tolerated [44]. A first in human phase 1 clinical trial was performed in patients with NSCLC (Table 1). The cohort included MET amplified, high c-Met, and high phospho-c-Met patients. No response was observed in patients with high c-Met; however significant antitumor response at 570 mg/m^2^ was observed in patients with MET amplification [33].

Tepotinib (EMD1214063) is a c-Met inhibitor with ≥1000 fold selectivity for c-Met as compared to other kinases. It inhibited both HGF-dependent and HGF-independent c-Met phosphorylation in vitro in lung and gastric cancer cell lines and showed tumor regression in xenografts model [45]. Tepotinib has been shown to overcome the acquired resistance to first generation EGFR TKIs in NSCLC with T790M mutation, displaying complete regression in xenograft studies when combined with rocelitinib, a third generation EGFR TKI that targets the T790M mutation [46]. Phase 1b/2 trial of tepotinib combined with gefitinib is on ongoing clinical study (NCT01982955) to evaluate the efficacy in terms of progression-free survival in advanced lung cancer (Table 1). Tepotinib is also being evaluated in a phase II single arm clinical trial in patients with advanced (stage III/IV) NSCLC harboring MET exon 14 skipping mutation (NCT02864992, Table 1).

Capmatinib (INCB28060) was identified as a very potent selective competitive inhibitor for c-Met, inhibiting activity at picomolar concentrations and displaying ≥10,000 selectivity for c-Met compared to other kinases. Effective anti-proliferative and anti-apoptotic properties of capmatinib were observed in c-Met driven mouse tumor models [47]. In NSCLC cell lines made resistant to erlotinib through the addition of HGF, capamitinib could re-sensitize cells to erlotinib [48]. Clinical studies are ongoing with exon 14 mutation or MET amplification (Table 1) to establish safety and pharmacokinetics. 

## 7. Multi-Kinase Targeting

An approach to improve responses to TKIs in patients lacking specific genetic alterations is to broaden the kinases being targeted. A number of TKIs are being tested clinically with ability to block multiple receptors (Table 1). Foretinib (XL-880) inhibits several kinases including c-Met, RON, VEGFR2, KIT, TIE2 and PDGFR [32], suggesting it could block proliferation and be anti-angiogenic. Foretinib was tested in a phase I trial given with erlotinib in NSCLC patients who progressed after chemotherapy. Responses were seen in 17.8% of evaluable patients, including those without EGFR mutation. Baseline c-Met expression was associated with response, suggesting combining this multi-kinase inhibitor with an EGFR inhibitor could improve sensitivity to erlotinib in both EGFR mutant and wild type patients. Cabozantinib (XL-184) is another multi-kinase inhibitor that targets c-Met, VEGFR1, VEGFR2, VEGFR3, RET, TIE2, FLT-3 and KIT, so should block multiple pro-cancer signaling pathways [49]. It has significant oral bioavailability and blood-brain barrier penetration. It was found to be superior to erlotinib and to improve outcomes when combined with erlotinib in patients who lacked EGFR mutations, with acceptable toxicity [49]. 

The multi-kinase inhibitor glesatinib (MGCD265) targets c-Met, VEGFR1, VEGFR2, VEGFR3, TIE2 and RON [50]. It is currently being tested in clinical trials for NSCLC in combination with erlotinib and docetaxel. A Phase 2 trial of glesatinib in combination with the checkpoint blocker nivolumab in patients with advanced NSCLC previously treated with platinum doublet chemotherapy and a checkpoint inhibitor is ongoing (Table 1). Another agent that targets both c-Met and RON, BMS-777607, showed an acceptable safety profile in a phase 1 trial, and a RON biomarker, CTX, was down-modulated by the drug [30].

## 8. Biological Antagonists of HGF or c-Met

Several types of biological antagonists have been developed that either neutralize HGF, c-Met, or the HGF-c-Met interaction. These agents are in various stages of development and can be used as a monotherapy or in combination with other targeted therapies. HGF-competitive analogs compete with the ligand for receptor binding on the cell surface. They do not lead to c-Met signaling and cannot induce c-Met dimerization, while HGF neutralizing antibodies bind to the fully processed HGF molecule, preventing interaction of HGF with the receptor. c-Met competitive variants competitively displace HGF and do not cause dimerization of the receptor. Decoys of c-Met have also been produced that bind to intact c-Met or HGF to disrupt dimerization of native c-Met receptor [51]. 

The following biological antagonists have been, or are currently being, clinically evaluated:

Onartuzumab (MetMAb): Onartuzumab is a monoclonal antibody against c-Met that obstructs the binding of the HGF α-chain to its c-Met ligand binding domain [52]. After safety evaluation [53], a phase II trial showed increased efficacy of onartuzumab in combination with erlotinib compared to erlotinib alone n patients positive for c-Met protein by immunohistochemical evaluation [54,55]; however further testing in a phase III trial was discontinued for lack of efficacy in this setting, in which the combination with erlotinib showed shorter survival [56]. 

Emibetuzumab (LY2875358): Emibetuzumab is a bivalent antibody raised against c-Met that blocks HGF binding to c-Met, preventing signaling. Unlike onartuzumab, it leads to internalization and degradation of c-Met. In a study using mouse xenograft models, emibetuzumab blocked both HGF-dependent and -independent tumor growth [57]. A phase I clinical trial with emibetuzumab alone or in combination with erlotinib was carried out in patients with NSCLC [58]. 23 patients received emibetuzumab alone, one patient experienced a partial response (4.3%) and five patients (21.7%) experienced stable disease. Out of the 14 NSCLC patients receiving combination treatment, two patients experienced a partial response (14.3%) and four (28.6%) had stable disease. Comparing emibetuzumb with and without erlotinib is now being tested in a phase 2 trial, and it is also currently being tested in a phase 1 trial in combination with an anti-VEGFR2 antibody, ramucirumab (Table 1).

LY3164530 is a bispecific monoclonal antibody that binds and degrades both c-Met and EGFR, which has shown strong ability to inhibit signaling from both receptors. In a xenograft model [59], LY3164530 had more anti-tumor effect in comparison to emibetuzumab and cetuximab, and was effective against NSCLC resistant to EGFR inhibitors [60]. It is now in phase 1 testing (Table 1). 

JNJ-61186372: The anti-tumor activity of JNJ-61186372, a c-Met and EGFR bispecific antibody, was investigated in in vitro and in vivo studies involving NSCLC tumor models, and showed strong ability to reduce tumor growth [61,62]. The safety and efficacy of JNJ-61186372 are currently being evaluated in a Phase 1 study in NSCLC (Table 1), to determine dosing for phase 2 studies and to identify any dose limiting toxicities. The study is scheduled for completion in 2020.

SAIT301: This is a humanized monoclonal antibody that targets the alpha chain of the extracellular domain of c-Met [63], preventing HGF binding. In addition, SAIT301 causes c-Met internalization that leads to degradation, which enhances the blockade of c-Met signaling. [63]. There is an ongoing phase I clinical study for patients with c-Met-positive solid tumors, using immunohistochemistry to detect positive c-Met staining (Table 1).

ABT-700 (h224G11): The anti-c-Met monoclonal antibody ABT-700 has anti-tumor effects in lung cancer xenografts with amplification of *MET* gene [64]. An ongoing phase I study of ABT-700 alone or in combination with one of three standard-of-care regimens is comparing the efficacy of monotherapy v/s combinational therapy in advanced solid tumors with *MET* gene amplification and/or c-Met overexpression (Table 1). 

Rilotumumab (AMG-102): Rilotumumab was the first HGF inhibitor to reach phase 3 clinical testing. It binds to the HGF β-chain, inhibiting HGF binding to c-Met [65]. Rilotumumab showed tolerability in the phase 1 clinical trial [66]. In the phase 2 study, addition of rilotumumab to capecitabine, cisplatin and epirubicin led to an increase in progression free survival and overall survival in *MET*-positive patients with adenocarcinoma [66]. However, there were two phase 3 clinical trials that showed negative results. For example, the RILOMET-1 study was stopped early (in 2014) because of lower efficacy, higher toxicity, shorter overall survival and lack of specific effects in NSCLC patients with *MET* amplification [67]. Rilotumumab was evaluated in combination with chemotherapy for small cell lung cancer. Overall survival was better with rilotumumab (10.8 months in placebo arm vs. 12.2 months in the rilotumumab arm) [34]. Rilotumumab was also tested in combination with erlotinib in a phase 1/2 study in NSCLC patients unselected for EGFR mutations status; the combination was found to have an acceptable safety profile. The disease control rate (DCR) for all patients was 60%. Among patients with wild type EGFR, the DCR was 60.6% and median overall survival was 7.0 months (90% CI, 5.6–13.4 months) [68], suggesting that blocking HGF in combination with an EGFR TKI might improve efficacy in the EGFR wild-type population.

Ficlatuzumab (AV-299): Ficlatuzumab is a humanized anti-HGF neutralizing antibody. In a phase I trial, ficlatuzumab alone had a maximum tolerance dose of 20 mg/kg in patients with NSCLC. The toxicities observed were low grade [69]. There was an increase in circulating HGF observed in patients after ficlatuzumab treatment, compared to the basal levels, suggesting a rebound effect occurs that could limit efficacy [70]. Results from a phase II study compared the efficacy of ficlatuzumab and gefitinib in combination versus gefitinib as monotherapy in Asian patients with lung adenocarcinoma. There was no significant difference in response rate observed in monotherapy (40%) compared to combination therapy (43%), or in progression-free survival (4.7 months in monotherapy vs. 5.6 months in combinational therapy) [71]. However, patients who were classified as VeriStrat-poor (a test for erlotinib sensitivity) had better outcomes with the combination, and might benefit from ficlatuzumab.

TAK-701: This humanized monoclonal antibody against HGF was active in overcoming gefitinib resistance observed in *EGFR*-mutant human NSCLC cells [72]. During a phase I study in patients with advanced solid malignancies, TAK-701 had a good safety profile, with only low grade adverse effects [72]. YYB-101 is a neutralizing monoclonal antibody against HGF. It binds to the HGF α-chain and blocks c-Met activation and cell metastasis in vitro. It also has an anti-tumor effect in several xenografts [73,74]. There is an ongoing phase I clinical study in patients with advanced solid tumors.

ARGX-111 is a c-Met targeting human monoclonal that elicits antibody-dependent cellular cytotoxicity as part of its mechanism of action. A phase 1 study [37] showed a good safety profile and some activity in patients with c-Met abnormalities. MP0250 is a dual-specific antibody mimetic (a designed ankyrin repeat protein (DARPin^®^) that functions as a neutralizing protein for both VEGF and HGF [75]. It contains two human serum albumin antibody mimic sequences that flank an anti-HGF and an anti-VEGF sequence, producing a moiety that binds and neutralizes both VEGF and HGF [75]. The rationale for dual targeting is the observation of up-regulation of the VEGF pathway when c-Met is inhibited [75]. MP0250 has shown preclinical activity against human patient-derived xenografts with HGF expression [75], and is being tested in a phase 2 clinical trial in solid tumors (Table 1).

DN30: This c-Met antibody under development for clinical use acts through several novel mechanisms to disrupt c-Met signaling, including causing degradation of the receptor. The mechanism of action of DN30 involves down-regulation of c-Met, in which receptor bound to DN30 at the cell surface is removed by proteolytic cleavage, resulting in shedding of the extracellular domain [76]. The cleaved c-Met fragment acts as a decoy receptor because a functional HGF binding site is still present, and the cleaved portion is able to sequester free HGF, as well as dimerize with any intact active c-Met receptors remaining on the cell surface. Both these actions render c-Met inactive by preventing homodimerization of intact c-Met receptors, or by forming nonfunctional heterodimers [77,78]. By this dual mechanism, DN30 efficiently blocks both HGF binding and c-Met phosphorylation, showing activities found in both TKIs and neutralizing antibodies [79]. The combined effect is that DN30 can block both the HGF-dependent and HGF-independent pathways. It showed anti-cancer effects in both in vitro and in vivo models of tumors with addiction to the c-Met pathway [79]. 

## 9. Mechanisms of Resistance to Inhibitors of the HGF/c-Met Axis

Multiple gene mutations and mechanisms are known to contribute to resistance to HGF/c-Met pathway blockade, such as HER, BRAF and KRAS pathways, or mutations in c-Met [80,81]. For example, in preclinical studies, there was maintenance of downstream PI3K and MAPK signaling by emergence of a mutation in the c-Met activation loop (Y1230), avoiding an interaction with a c-MET TKI; activation of the EGFR pathway through secretion of transforming growth factor α also was a resistance mechanism [81]. NSCLC models showed that resistance to anti-MET agents was accompanied by upregulation of the Wnt and mTOR pathways [82]. Increased HGF secreted into the microenvironment can also overcome the action of anti-MET drugs and convert genetically altered constitutively active c-Met tumors into ligand-dependent tumors [83]. Proposed strategies that can be used to overcome acquired resistance in patients with basal sensitivity to HGF/c-Met pathway inhibition include adding inhibitors at different upstream and downstream levels of the pathway, adding an HGF neutralizing antibody to a c-Met targeting drug, and using upfront drug combinations to circumvent bypass mechanisms. 

## 10. Role of HGF-c-Met in Resistance to Other Therapies

The tumor microenvironment is engaged in resistance to molecular-targeted therapies. Stromal cells influence the action of cancer therapeutics, and stromal changes such as release of HGF can provide an alternate stimulus when other pathways are blocked. In a study using a RAF inhibitor, increased HGF secretion was identified as a prime mechanism for resistance [84]. Although the T790M second mutation in the mutant EGFR often occurs in NSCLC patients with acquired resistance to EGFR TKIs [85], HGF-dependent c-Met activation and constitutive activation by amplification of the *MET* gene are noted compensatory mechanisms that also are factors in acquired resistance in EGFR mutant NSCLC patients [86,87]. Alectinib, a selective anaplastic lymphoma kinase (ALK) TKI that lacks interaction with c-Met, has high activity in ALK mutant NSCLC patients; alcetinib treatment in ALK positive patients was associated with increased median progression free survival and high response rate [88]. However, many NSCLC patients eventually became resistant to alectinib and one common mechanism of acquired resistance to ALK TKIs is the secretion of HGF into the tumor microenvironment, leading to HGF-dependent c-Met signaling [89]. This is another example of how over-activity of the c-Met pathway is an important escape mechanism in targeted therapy resistance. 

## 11. Conclusions

Although the c-Met pathway is frequently overactive in NSCLC, inhibiting either the c-Met receptor itself or its ligand HGF has not proven effective as single therapy in unselected NSCLC patients. Clinical response to these agents has to date been largely restricted to NSCLC patients with genetic alterations in *MET*, such as amplification or exon 14 deletion. Some patients with acquired EGFR TKI drug resistance that involves up-regulation of the c-Met pathway also have responded. Acquired resistance that eventually develops after initial response to c-Met pathway inhibitors often involves activation of parallel signaling pathways or induction of HGF secretion. Several new classes of HGF/c-Met inhibitors may show expanded activity in patients with *MET* genetic alterations, and might also prove effective in NSCLC with overexpressed c-Met in the absence of genetic abnormality. These include the multi-kinase inhibitors that block c-Met as well as a range of other kinases; the kinase inhibitors and monoclonal antibodies against c-Met that also cause degradation of the c-Met protein; and the bivalent antibodies that can block several pathways simultaneously. The newer c-Met TKIs with improved c-Met selectivity compared to earlier agents most likely will only be active as single agents in the presence of *MET* oncogene addiction. Combinations of kinase inhibitors that target both upstream and downstream in the signaling pathway, or reduce parallel kinase signaling from other receptors, could improve clinical benefit, but toxicity has been a problem with these targeted combinations in the past. Combining a c-Met TKI or antibody with an HGF neutralizing antibody may improve efficacy. Other approaches to expand efficacy of HGF/c-Met targeting include combining these agents with inhibitors of the viability or function of stromal cells such as tumor-associated fibroblasts, endothelial cells, or macrophages, or using immunotherapy in combination with HGF/c-Met agents. Such strategies might also increase activity of these drugs in a wider range of patients who lack MET genetic abnormalities. 

## Figures and Tables

**Table 1:** Active or Recent Clinical Trials of hepatocyte growth factor (HGF)-MET Inhibitors in Lung Cancer or in Solid Tumors.

Agent(s) and Mechanism	Trial Phase	Endpoints	Patient Population/Indication	Study Design	Clinical Trial Identifier and Status Source: www.Clinicaltrials.gov
Capmatinib (INCB28060)c-Met ATP-competitive inhibitor	1	Safety, tolerability, PK	c-MET-dysregulated advanced solid tumors	Open Label, Dose Escalation Study of Tablet Formulation	NCT02925104 Status: Recruiting
Capmatinib	1	Safety	Malignant NSCLC with MET exon 14 skipping alteration	Capmatinib oral daily (50–740 mg/m^2^) 21-day cycles	NCT02750215 Status: Active, not recruiting
Cabozantinib (XL184) c-Met, VEGFR2, and RET ATP-competitive inhibitor	2	Efficacy	Advanced or metastatic solid tumors	All subjects start cabozantinib at 40 mg. Those who tolerate 40 mg for 2 cycles will escalate to 60 mg	NCT02101736 Status: Active, not recruiting
Cabozantinib (XL184)	2	Safety/efficacy	Advanced NSCLC, RET, ROS1, or NTRK fusion-positive	Initial dose of 60 mg orally daily for 28-day cycles	NCT01639508 Status: Recruiting
BMS-777607 (ASLAN002) RON and c-Met ATP-competitive inhibitor	1	Safety	Advanced or metastatic solid tumors	Oral daily doses of 100 mg, 200 mg, 300 mg, 450 mg, or 600 mg	NCT01721148 Status: Completed Safety profile acceptable Down-modulation of a RON biomarker (CTX) found [30]
Volitinib (HMPL-504) c-Met ATP-competitive inhibitor	1	Safety/efficacy	Advanced solid tumors	Oral tablet of 25 mg, 100 mg and 200 mg, once daily or 2 times a day	NCT01985555 Status: Active Patients with c-Met dysregulation showed responses [31]
Volitinib	1	Safety, PK, Efficacy	EGFR mutation-positive NSCLC patients who progressed on EGFR tyrosine kinase inhibitor	Volitinib at 600 or 800 mg orally once daily Gefitinib at 250 mg orally once daily	NCT02374645 Status: Active, not recruiting
Tepotinib (EMD1214063) c-Met ATP-competitive inhibitor plus Gefitinib (EGFR TKI)	2	Efficacy	Advanced NSCLC	Tepotinib at 300 or 500 mg orally once daily over a 21-day cycle Gefitinib at 250 mg orally once daily over a 21-day cycle	NCT01982955 Status: Active, not recruiting
Tepotinib	2	Efficacy/Safety	Advanced NSCLC with MET Exon 14 Skipping Alterations	500 mg once orally daily in 21-day cycles	NCT02864992 Status: Recruiting
Foretinib (GSK1363089) multi-kinase ATP-competitive inhibitor of c-Met and VEGFRs plus Erlotinib (EGFR TKI)	1	Safety	Previously treated advanced NSCLC unselected for EGFR genotype	150 mg erlotinib once daily and 30–45 mg foretinib added on day 15 of cycle 1	NCT01068587 Status: Completed Responses seen in 17.8% of evaluable patients. Baseline c-Met expression associated with response. Incremental toxicity seen [32]
Glesatinib (MGCD265) c-Met and multiple kinase ATP-competitive inhibitor plus Nivolumab (PD-1 blocker)	2	Safety/Efficacy	Advanced NSCLC, previously treated with platinum doublet chemotherapy and a checkpoint inhibitor	Twice daily oral glesatinib (two doses tested) Nivolumab 240 mg IV every 2 weeks	NCT02954991 Status: Recruiting
SAR125844 c-Met selective ATP-competitive inhibitor	1	Safety, PK, Preliminary Efficacy	Advanced solid tumors with MET amplification or phospho-c-Met expression	Escalating doses (50–740 mg/m^2^) given IV weekly for 6 weeks or until progression	NCT02435121 Status: Completed Drug was well tolerated and anti-tumor activity was observed only in MET amplified patients [33]
Emibetuzumab (LY2875358) anti-c-Met bivalent antibody plus Ramucirumab (anti-VEGFR2 antibody)	1	Safety	Advanced or metastatic solid tumors	Dose escalation of IV emibetuzumab, in combination with a fixed dose of IV ramucirumab on days 1 and 15 of every 28 day cycle	NCT02082210 Status: Active, not recruiting
Emibetuzumab Plus Erlotinib	22	Efficacy	NSCLC with activating EGFR mutations	Lead In: 8 weeks of oral daily Erlotinib, 150 mg Randomization: Emibetuzumab (20 mg) given IV on Days 1 and 15 of 28-day cycles, with and without Erlotinib.	NCT01897480 Status: Active, not recruiting
Rilotumumab (AMG 102) Human IgG2 monoclonal neutralizing antibody to HGF	22	Efficacy	Stage IV SCLC	Rilotumumab 15 mg/kg given with etoposide and carboplatin or cisplatin	NCT00791154 Status: Completed Outcomes not improved although low HGF levels associated with improved survival [34]
YYB-101 Neutralizing humanized monoclonal Ab against HGF	11	Safety/Efficacy	Solid tumors	Increasing dose (0.3 mg/kg to 5 mg/kg), IV on Day 1 and Day 29, followed by every 2 weeks. Dose-expansion cohort: MTD (or RP2D), IV infusion every 2 weeks	NCT02499224 Status: Recruiting
Ficlatuzumab (AV-299) humanized IgG1 monoclonal antibody against HGFplus Gefitinib	1b	Safety/Efficacy	Asian NSCLC patients, unselected for EGFR mutation	Ficlatuzumab 10 mg/kg or 20 mg/kg IV on days 1 and 15 of a 28 day cycle. Gefitinb 250 mg orally daily	NCT Status: Completed Dose-related activity seen in patients with no prior EGFR TKI treatment, some in EGFR WT patients [35]
TAK-701 humanized monoclonal antibody to HGF	11	Safety/Efficacy	Advanced solid tumors	2, 5, 10, or 20 mg/kg IV. Cycle 1: single dose at 2x the dose assignment; Cycle 2 and beyond: dose once every two weeks	NCT00831896 Status: Completed TAK-701 was well tolerated [36]
SAIT301 Monoclonal Ab against c-Met that induces c-Met degradation	11	Safety/Efficacy	Solid tumors	8 cohorts comprised of 3 to 6 subjects each. SAIT301 will be administered according to a 3 + 3 design	NCT02296879 Status: Completed, No results posted
LY3164530 c-Met/EGFR bispecific antibody	11	Safety/Efficacy	Solid tumors	LY3164530 in escalating dose cohorts given IV once on Days 1, 8, 15, and 22 of a 28-day cycle	NCT02221882 Status: Completed, No results posted
JNJ-61186372 c-Met/EGFR bispecific antibody	11	Safety/Efficacy	NSCLC	Increasing dose levels for 28 day cycles. The dose will be escalated until the MDT	NCT02609776 Status: Recruiting
ARGX-111 c-Met-targeting human monoclonal Ab that activates antibody-dependent cellular cytotoxicity	11	Safety/Efficacy	c-MET-overexpressing cancer	Doses given were-0.3 mg/kg, 1.0 mg/kg, 3.0 mg/kg and 10 mg/kg	NCT02055066 Status: Completed Good safety profile, some activity in patients with c-Met abnormalities [37]
MP0250 Dual anti-HGF/anti-VEGF antibody mimetic	22	Safety/Efficacy	Advanced solid tumors	IV infusion at up to six dose levels, every other week for up to 24 infusions	NCT02194426 Status: Active, Not recruiting
ABT-700 c-Met monoclonal antibody	11	Safety/efficacy	Advanced solid tumors with MET amplification or overexpression	IV infusion at escalating doses in 21-day cycles ABT-700 will also be given in combination with other therapies in 3 cohorts	NCT01472016 Status: Completed No results posted
